# Supplementary material for: HIVprotI: an integrated web based platform for prediction and design of HIV proteins inhibitors
Source: J Cheminform. 2018 Mar 9;10:12. doi: 10.1186/s13321-018-0266-y (PMC5845081; doi:10.1186/s13321-018-0266-y)
Supplement: Supplementary file 2 — Additional file 2. Source code of HIVProtI web server. [file 13321_2018_266_MOESM2_ESM.zip › HIVprotI_Source-code/hivprotI/weka-3-6-6/doc/weka/core/pmml/Function.html]

Function


---


|  |  |  |
| --- | --- | --- |
| **Overview** \| **Package** \| **Class** \| **Tree** \| **Deprecated** \| **Index** \| **Help** \| **Weka's home** | |  |
| **PREV CLASS**   **NEXT CLASS** | **FRAMES**    **NO FRAMES**     **All Classes** |  |
| SUMMARY: NESTED \| FIELD \| CONSTR \| METHOD | DETAIL: FIELD \| CONSTR \| METHOD |  |


---


## weka.core.pmml Class Function

```
java.lang.Object
  weka.core.pmml.Function
```

**All Implemented Interfaces:**: java.io.Serializable

**Direct Known Subclasses:**: BuiltInArithmetic, BuiltInMath, BuiltInString, DefineFunction

---

``` public abstract class Function extends java.lang.Object implements java.io.Serializable ```

Abstract superclass for PMML built-in and DefineFunctions.

**Version:**
:   $Revision 1.0 $

**Author:**
:   Mark Hall (mhall{[at]}pentaho{[dot]}com)

**See Also:**: Serialized Form

---

**Constructor Summary**

`Function()`


| **Method Summary** | |
| --- | --- |
| `static Function` | `getFunction(java.lang.String name)`             Get a built-in PMML Function. |
| `static Function` | `getFunction(java.lang.String name, weka.core.pmml.TransformationDictionary transDict)`             Get either a function. |
| `java.lang.String` | `getName()` |
| `abstract  Attribute` | `getOutputDef()`             Get the structure of the result produced by this function. |
| `abstract  java.lang.String[]` | `getParameterNames()`             Returns an array of the names of the parameters expected as input by this function. |
| `abstract  double` | `getResult(double[] incoming)`             Get the result of applying this function. |
| `abstract  void` | `setParameterDefs(java.util.ArrayList<Attribute> paramDefs)`             Set the structure of the parameters that are expected as input by this function. |
| `java.lang.String` | `toString()` |
| `java.lang.String` | `toString(java.lang.String pad)` |

**Methods inherited from class java.lang.Object**

`equals, getClass, hashCode, notify, notifyAll, wait, wait, wait`

**Constructor Detail**

### Function

```
public Function()
```


**Method Detail**

### getName

```
public java.lang.String getName()
```

---


### getParameterNames

```
public abstract java.lang.String[] getParameterNames()
```

:   Returns an array of the names of the parameters expected
    as input by this function. May return null if this function
    can take an unbounded number of parameters (i.e. min, max, etc.).

    :   **Returns:**: an array of the parameter names or null if there are an unbounded number of parameters.

---


### setParameterDefs

```
public abstract void setParameterDefs(java.util.ArrayList<Attribute> paramDefs)
                               throws java.lang.Exception
```

:   Set the structure of the parameters that are expected as input by
    this function. This must be called before getOutputDef() is called.

    :   **Parameters:**: `paramDefs` - the structure of the input parameters **Throws:**: `java.lang.Exception` - if the number or types of parameters are not acceptable by this function

---


### getOutputDef

```
public abstract Attribute getOutputDef()
```

:   Get the structure of the result produced by this function.

    :   **Returns:**: the structure of the result produced by this function.

---


### getResult

```
public abstract double getResult(double[] incoming)
                          throws java.lang.Exception
```

:   Get the result of applying this function.

    :   **Parameters:**: `incoming` - the arguments to this function (supplied in order to match that of the parameter definitions **Returns:**: the result of applying this function. When the optype is categorical or ordinal, an index into the values of the output definition is returned. **Throws:**: `java.lang.Exception` - if there is a problem computing the result of this function

---


### getFunction

```
public static Function getFunction(java.lang.String name)
```

:   Get a built-in PMML Function.

    :   **Parameters:**: `name` - the name of the function to get. **Returns:**: a built-in Function or null if the named function is not known/supported.

---


### getFunction

```
public static Function getFunction(java.lang.String name,
                                   weka.core.pmml.TransformationDictionary transDict)
                            throws java.lang.Exception
```

:   Get either a function. Built-in functions are queried first, and then
    DefineFunctions in the TransformationDictionary (if any).

    :   **Parameters:**: `name` - the name of the function to get.: `transDict` - the TransformationDictionary (may be null if there is no dictionary). **Returns:**: the function **Throws:**: `java.lang.Exception` - if the named function is not known/supported.

---


### toString

```
public java.lang.String toString()
```

:   **Overrides:**: `toString` in class `java.lang.Object`

---


### toString

```
public java.lang.String toString(java.lang.String pad)
```


---


|  |  |  |
| --- | --- | --- |
| **Overview** \| **Package** \| **Class** \| **Tree** \| **Deprecated** \| **Index** \| **Help** \| **Weka's home** | |  |
| **PREV CLASS**   **NEXT CLASS** | **FRAMES**    **NO FRAMES**     **All Classes** |  |
| SUMMARY: NESTED \| FIELD \| CONSTR \| METHOD | DETAIL: FIELD \| CONSTR \| METHOD |  |


---
